# Comparison of fish oil supplements and corn oil effects on serum lipid profile: a systematic review and meta-analysis of randomized controlled trials

**DOI:** 10.1186/s13643-023-02426-8

**Published:** 2024-02-05

**Authors:** Payam Safaei, Ghazal Bayat, Afsaneh Mohajer

**Affiliations:** 1 Division of Food Safety and Hygiene, Department of Environmental Health Engineering, School of Public Heath, Tehran University of Medical Sciences, Tehran, Iran; 2 Department of Clinical Pathology, Faculty of Veterinary Medicine, University of Tehran, Tehran, Iran

**Keywords:** Fish oil, Corn oil, TC, LDL, HDL, TG

## Abstract

**Background:**

The present study aimed to investigate the effects of fish oil supplements compared to corn oil on serum lipid profiles by performing a meta-analysis of randomized controlled trials (RCTs).

**Methods:**

Online databases including PubMed, Web of Science, and Scopus were searched until 30 December 2022. Pooled effect sizes were reported as the weighted mean difference (WMD) with 95% confidence intervals (CI). The Cochrane Collaboration’s risk-of-bias tool was utilized to evaluate the quality of the studies. Lipid parameters, including triglycerides (TG), total cholesterol (TC), low-density lipoprotein cholesterol (LDL), and high-density lipoprotein cholesterol (HDL), were assessed in the meta-analysis.

**Results:**

Overall, 16 eligible trials were included in this systematic review and meta-analysis. The results revealed that the fish oil supplements significantly reduced TG (*WMD*: − 25.50 mg/dl, 95% *CI*: − 42.44, − 8.57, *P* = 0.000) levels compared to corn oil. Also, in this study, fish oil supplements had a positive and significant effect on HDL (*WMD*: 2.54 mg/dl, 95% *CI*: 0.55, 4.52). There were no significant changes in TC and LDL.

**Conclusions:**

Our findings showed the effects of fish oil supplements on reducing TG and increasing HDL-c compared to corn oil. Further larger and well-designed RCTs are required to confirm these data.

## Introduction

Cardiovascular diseases (CVDs) are a group of diseases that affect the heart and blood vessels [1]. CVD is the first cause of mortality in the world, responsible for nearly one-third of all deaths [2, 3]. The risk of CVDs is thought to be increased by conditions known as cardiometabolic risk factors (CMR), which include dyslipidemia, hypertension, diabetes, overweight, abdominal obesity, and inflammation [4]. Dyslipidemia is regarded as the most important risk factor that increases the possibility of CVDs. It is described by elevated total cholesterol (TC), plasma triglycerides (TG), low-density lipoprotein cholesterol (LDL-c), and low levels of plasma high-density lipoprotein cholesterol (HDL-c) [5]. Existing evidence indicates that controlling these lipids can effectively reduce the risk of cardiovascular disease [6]. Medication is the usual cure, and lifestyle changes are the important strategies in the management of dyslipidemia. Diet modification can have powerful effects on reducing the need for pharmacologic interventions and their side effects. In particular, oils, due to their various fatty acid compositions, including saturated fatty acids (SFAs), trans fatty acids, monounsaturated fatty acids (MUFAs), and polyunsaturated fatty acids (PUFAs), have different effects on the lipid profile and play an important role in the development of CVD [7]. While the fatty acid content of oils may estimate the influence of fats on serum lipid profiles [8], knowing which type of dietary oil has the most effects on lipoproteins still needs further investigation. Recent evidence has revealed that monounsaturated fatty acid (MUFA) intake promotes a healthy blood lipid profile [9], glycemic control [10], and insulin resistance [11]. In addition, the consumption of vegetable and fish oils, which are rich in PUFAs, has been linked to potential cardioprotective, lipid profile, and blood glucose control benefits [12, 13]. Therefore, MUFAs as well as PUFAs have positive effects on human health by lowering the risk of metabolic events. In this regard, the consumption of fish and corn oils, which are rich in MUFAs and PUFAs, respectively, has been recommended. The cardioprotective benefits of these oils have been reported in several studies [14–16]. Fish oil is a rich source of omega-3 polyunsaturated fatty acids, especially eicosapentaenoic acid (EPA) and docosahexaenoic acid (DHA), which, compared to types of omega-3 found in plants, are healthier choices for consumption [17]. Fish oil supplementation has been shown to improve multiple risk factors for heart disease [18]. Moreover, it has been associated with some beneficial effects on glycemic control, blood pressure, and the inflammatory response [19]. Hence, there has been increasing attention paid to taking a fish oil supplement in recent years. Corn oil is another oil that has become popular for cooking applications recently. This vegetable oil comprises a high concentration of polyunsaturated omega-6 fat, especially linoleic acid [20]. Compared with other oils, corn oil is one of the richest dietary sources of phytosterols and tocopherols. *β*-sitosterol (63–70%) and *γ*-tocopherol (68–89%) are the main types of phytosterol and tocopherol in corn oil, respectively. In some studies, corn oil has been linked to improved plasma lipids, including serum total cholesterol and triglyceride levels, which may be due to the high level of phytosterols in this oil and thus reduce the risk of heart disease. Also, it has been demonstrated that *γ*-tocopherol displays efficacy against DNA damage, blood pressure, and diabetes [21, 22].

Although previous randomized controlled trials (RCTs) evaluated the impacts of fish oil on lipid profiles and cardiovascular factors, its effects compared to corn oil in the subgroup analysis were still inconsistent. Given the fatty acid content and wide consumption of fish and corn oils, there are many claims that describe their effects on improving blood lipids. It is undoubtedly necessary to conduct a meta-analysis to summarize the evidence and comprehensively compare these two oils, paying attention to the higher percentage of omega-3 in fish oil. To the best of our knowledge, there is no meta-analysis in this area. Therefore, the purpose of this study was to perform a systematic review and meta-analysis of randomized controlled trials (RCTs) to compare the effects of fish oil supplements and corn oils on the lipid profile.

## Methods

### Search strategy

The current study was carried out according to the Preferred Reporting Items for Systematic Reviews and Meta-Analyses (PRISMA) guidelines [23]. PubMed, Scopus, and Web of Science databases were searched until December 30, 2022. Randomized controlled trials (RCTs) examined the effects of fish oil and corn oil consumption on serum lipid profiles. We used a combination of the following keywords to search the databases: (("low-density lipoprotein") OR (LDL*) OR ("total cholesterol") OR (TC) OR ("high-density lipoprotein") (HDL*) OR (triglyceride*) OR (TG) OR ("polyunsaturated fatty acids") OR (eicosapentaenoic acid) OR (EPA*) OR (docosahexaenoic acid*) OR (DHA*) OR (lipoprotein *) OR ("lipid profile") OR (Lipid*) OR ("cardiovascular disease") OR ("heart disease") OR (hypercholesterolemia*)) AND ((corn oil*) OR ("maize oil") OR ("fish oil") NOT ((rat) OR (mouse) OR (animal*)). The references of related papers were checked to identify additional studies that were not found through online searches.

### Study selection and eligibility criteria

The review of literature involved a meticulous examination of the titles, abstracts, and full texts by two independent experts with the aim of identifying potentially relevant articles. Specifically, clinical trials that presented original data on the impacts of fish oil and corn oil interventions on serum lipid factors, including TC, LDL-c, HDL-c, or TG, were taken into consideration. The inclusion criteria established were that the studies must have been published in English, have full text available, feature corn oil as the control group, provide a comparison group, and have been published until December 30, 2022. Conversely, the exclusion criteria comprised studies that had another oil as the control group, studies without complete information or a control group, and studies presented in the form of illegible graphs, animal and in vitro studies, reviews and meta-analyses, and articles with nonclinical trial designs.

### Data extraction

After reviewing the articles, all the required information was extracted by two researchers using a predefined screening form. We extracted the data from each selected study, including the first author’s name, year of publication, country, age, sex, baseline body mass index (BMI), study design, sample size, type and dose of intervention and control group, duration of study, and state of health. Moreover, the mean changes of TG, TC, HDL-c, and LDL-c from baseline to the end of the study and their standard deviations (SDs) were also extracted.

### Quality assessment

The quality of included studies was assessed independently by two authors, according to the Cochrane Collaboration’s tool [24], which is composed of the following criteria: (1) random sequence generation, (2) allocation concealment, (3) blinding of participants and personnel, (4) blinding of outcome assessment, and (5) incomplete outcome data, selective outcome reporting, and other biases based on the domains mentioned, and each study was classified as having a high bias risk, a low bias risk, and an unclear bias risk.

### Data synthesis and analysis

The effects of oils on the change in outcome factors were calculated. The weighted mean difference (WMD) with a 95% confidence interval (CI) between intervention and control groups was applied to determine effect sizes. The mean change of variables and the relevant standard deviations (SD) were elicited for analysis. In those studies that did not provide mean and SD changes from baseline, the mean of variables before and after intervention and the SD changes were yielded using a correlation coefficient r (*r* = 0.5) [25]. Regarding those studies that reported standard error (SE), the following formula was used to calculate SD: SD = SE × √n (*n* = number of subjects). The reported concentration of outcomes was converted into the usual unit (mg/dl) in the meta-analysis. The heterogeneity of the included studies was assessed by the chi-square (*I*^2^) index and the Cochrane Q test with a *p* < 0.05. An *I*^2^ greater than 50% was considered significant heterogeneity. Subgroup analyses based on age, amount of consumed oil, health status, and duration of treatment were conducted to determine the source of heterogeneity between studies. Potential publication bias was also checked through Egger’s funnel plots. Meta-analysis of data was performed using STATA 14.0 (Statistical Software, College Station, TX, USA). Statistical significance was defined by *p*-values < 0.05.

## Results

### Study selection

**Fig. 1:**
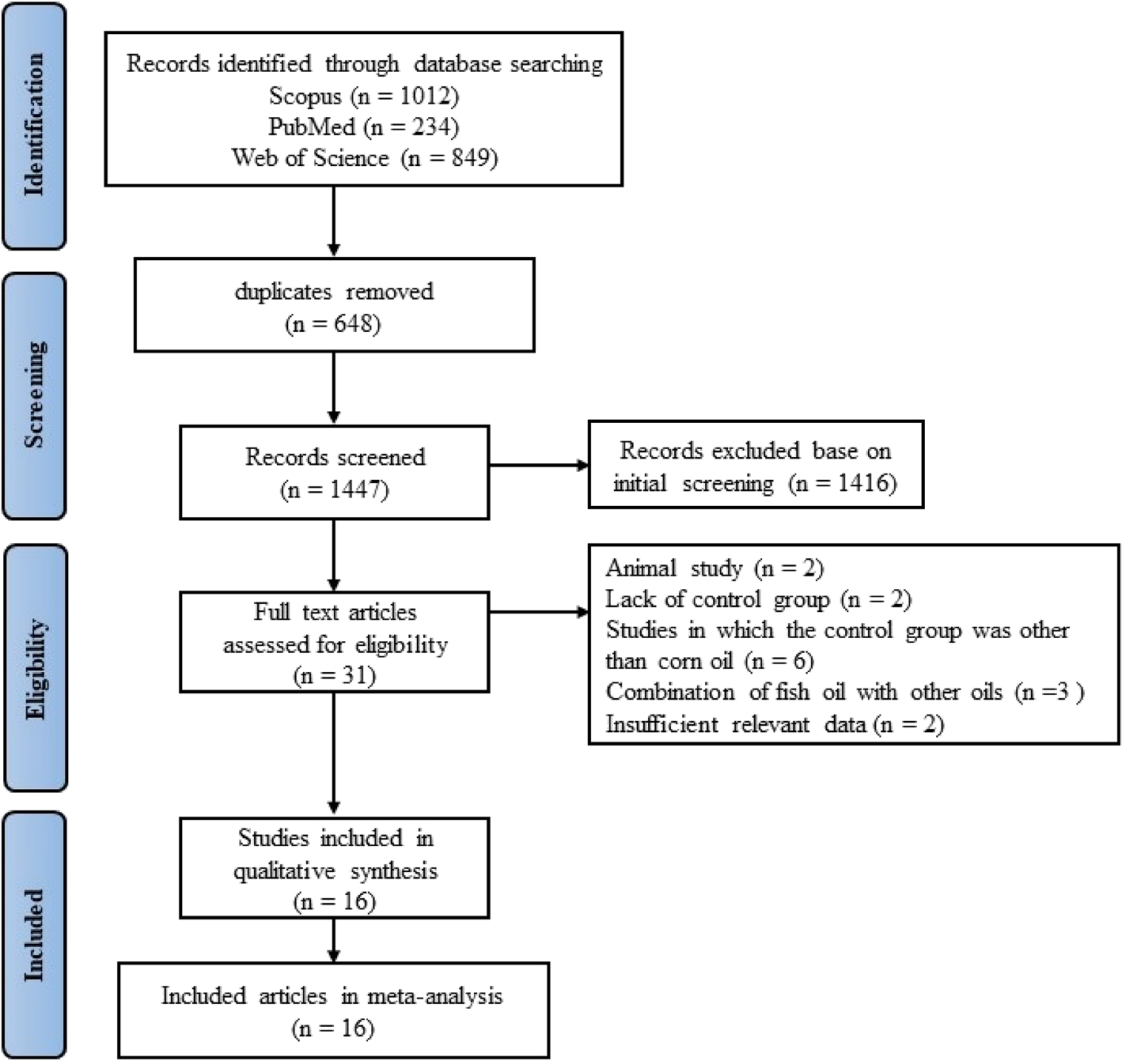
Flow chart of the study selection process

The flow diagram of the article selection process is presented in Fig. 1. In the primary search, 2095 potential records were identified from the literature search, and 648 articles were excluded because of duplication. After screening titles and abstracts and removing irrelevant studies, 31 articles were retained for further assessment. Of these articles, 15 were removed for the following reasons: animal studies (*n* = 2), studies that did not have a control group (2), studies in which the control group was other than corn oil (*n* = 6), articles that used the effects of fish oil in combination with other oils and/or interventions (*n* = 3), and without complete information about the mean and standard deviation of the control and/or intervention groups (*n* = 2). Finally, 16 articles were included in the meta-analysis.

### Study characteristics

The main characteristics of the included studies are displayed in Table 1. A total of 491 participants in the 16 randomized clinical trials examined the effects of fish oil on the serum lipid profile compared with corn oil as a control. Overall, the age range of participants and the intervention duration were 14 to 65 years and 28 to 180 days, respectively. In terms of the health status of the participants, in one study, there were healthy subjects [25], five studies involved patients with lipid disorders [26–30], four studies involved type 2 diabetes mellitus [31–34], two studies included patients with kidney disorders [35, 36], and other trials included patients with heart disease [37], fatty liver disorder [38], and high blood pressure [39]. All the articles were published between 2007 and 2019. These trials were conducted in different countries, including Iran [36], China [32, 38, 39], the USA [30, 34, 35], Israel [26], Norway [37], Australia [27], Germany [28], Canada [25], Denmark [31, 33, 40], and England [29]. Out of 16 studies, 16 RCTs investigated the intervention’s efficacy on TC, 15 on TG, 16 on HDL-c, and 13 on LDL-c.

**Table 1:** Main characteristics of included trials

Author (year)	Country	Population	Age (year)	Sample size Case/control	Dose	Duration (days)	Outcomes	Results
Petersen et al. (2002) [31]	Denmark	Type 2 diabetes	62.8	20/22	4	56	TC, TG, HDL-c, LDL-c	TG changed significantly
Qin et al. (2015) [38]	China	NAFLD	45.15	36/34	4	90	TC, TG, HDL-c, LDL-c	TC and TG changed significantly
Bowden et al. (2009) [35]	USA	Dialysis	60	44/43	2	180	TC, TG, HDL-c, LDL-c	HDL-c and LDL-c changed significantly
Parviz Khajehdehi (2000) [36]	Iran	Dialysis	33.15	15/15	4.5	60	TC, TG, HDL-c, LDL-c	TG and LDL-c changed significantly
Wang et al. (2017) [32]	China	Type 2 diabetes	65.4	49/50	4	180	TC, TG, HDL-c, LDL-c	TG and HDL-c changed significantly
Bitzur et al. (2010) [26]	Israel	Hyperlipidemia	49.6	34/33	4	84	TC, TG, HDL-c, LDL-c	TG changed significantly
Pedersen et al. (2003) [33]	Denmark	Type 2 diabetes	63	23/21	4	56	TC, TG, HDL-c	TG and HDL-c changed significantly
Nilsen et al. (2001) [37]	Norway	MI	64	123/123	4	180	TC, TG, HDL-c	TC, TG, HDL-c changed significantly
Chan et al. (2003) [27]	Australia	Dyslipidemic	54.5	12/12	4	42	TC, TG, HDL-c, LDL-c	TG changed significantly
Schmidt et al. (2012) [28]	Germany	Dyslipidemic	41	9/8	3.05	84	TC, TG, HDL-c, LDL-c	TG and HDL-c changed significantly
Ramprasath et al. (2013) [25]	Canada	Healthy	28.23	8/8	3	28	TC, TG, HDL-c, LDL-c	Not changed significantly
Lee et al. (2014) [34]	USA	Type 2 diabetes	58	16/21	NR	56	TC, TG, HDL-c, LDL-c	TG and HDL-c changed significantly
Borthwick et al. (1998) [29]	England	Hyperlipidemia	53.4	29/26	4	84	TC, HDL-c	TG changed significantly
Thusgaard et al. (2009) [40]	Denmark	HIV	45	25/23	3.6	84	TC, TG, HDL-c, LDL-c	TG changed significantly
Yang et al. (2019) [39]	China	Hypertensive	56.7	35/34	4	84	TC, TG, HDL-c, LDL-c	Not changed significantly
Gidding et al. (2014) [30]	USA	Hyperlipidemia	14.1	19/18	4	56	TC, TG, HDL-c, LDL-c	LDL-c changed significantly

*NR* not report

### Quality assessment

**Fig. 2:**
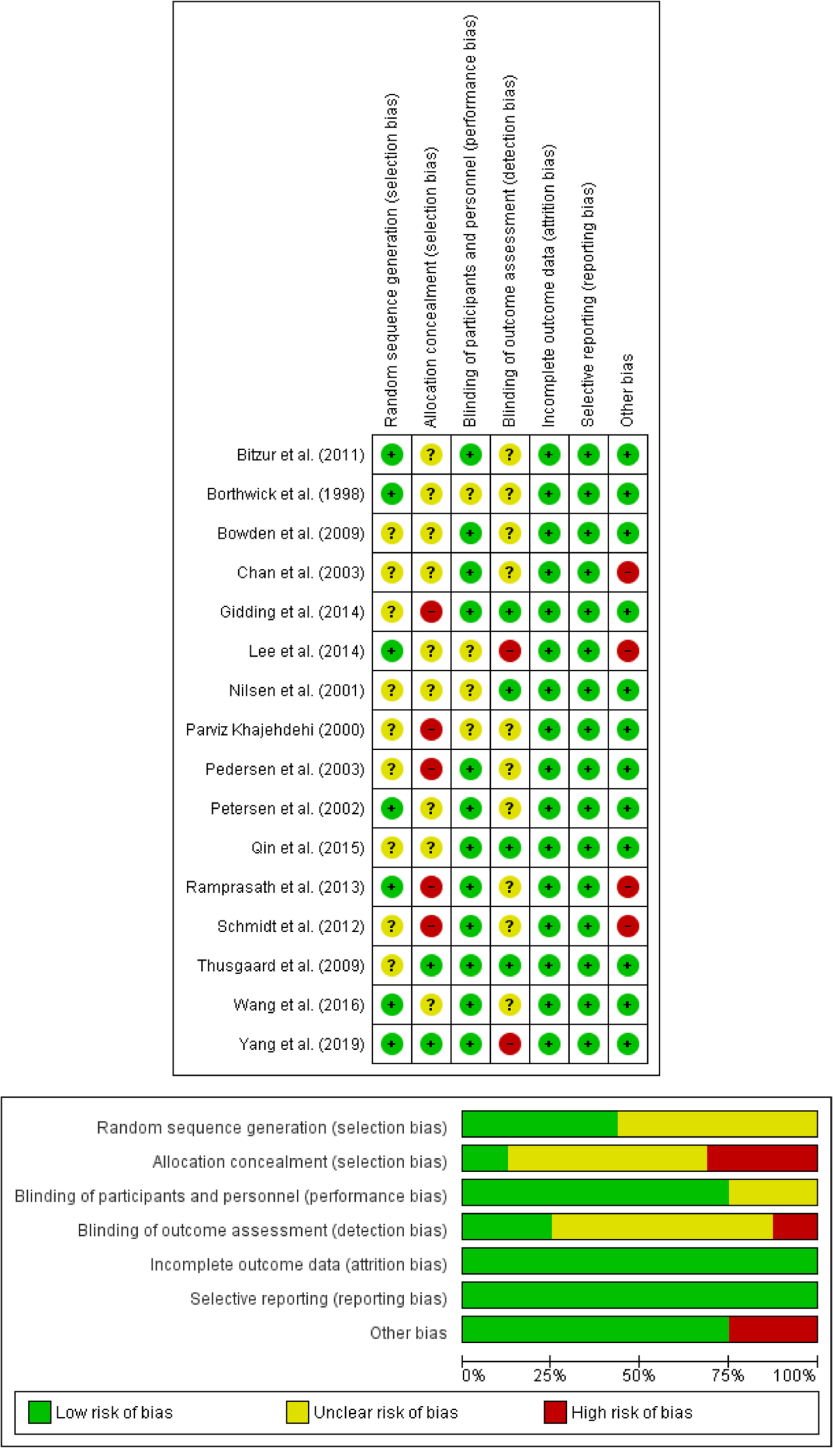
Cochrane risk-of-bias assessment

The quality assessment of included studies is shown in Fig. 2. As shown in Fig. 2, seven studies were assessed as having a low risk of bias in the random sequence generation [25, 26, 29, 31, 32, 34, 39], and other studies showed an unclear risk of bias [27, 28, 30, 33, 35–38, 40]. Five trials showed a high risk of bias for allocation concealment [25, 28, 30, 31, 36], nine did not provide specific methods for this operation [26, 27, 29, 31, 32, 34, 35, 37, 38], and two had a low risk of bias [39, 40]. There were four studies [29, 34, 36, 37] using a double-blind design that were classified as having an unclear risk of bias for the blinding of the participants and personnel, and the others showed a low risk of bias [25–28, 30–33, 35, 38–40]. Regarding blinding of outcome assessment, 4 studies showed a low risk of bias [30, 37, 38, 40], 10 had an unclear risk [25–29, 31–33, 35, 36], and only 2 showed a high risk of bias [34, 39]. All studies provided complete outcome data for analysis [25–40]. Also, all studies were judged to have a low risk of bias in selective reporting [25–40]. Four studies did not report other measures of quality, such as dose [27], or had imprecisions [25, 28, 34] (Fig. 2).

### Meta-analysis

#### TC

**Fig. 3:**
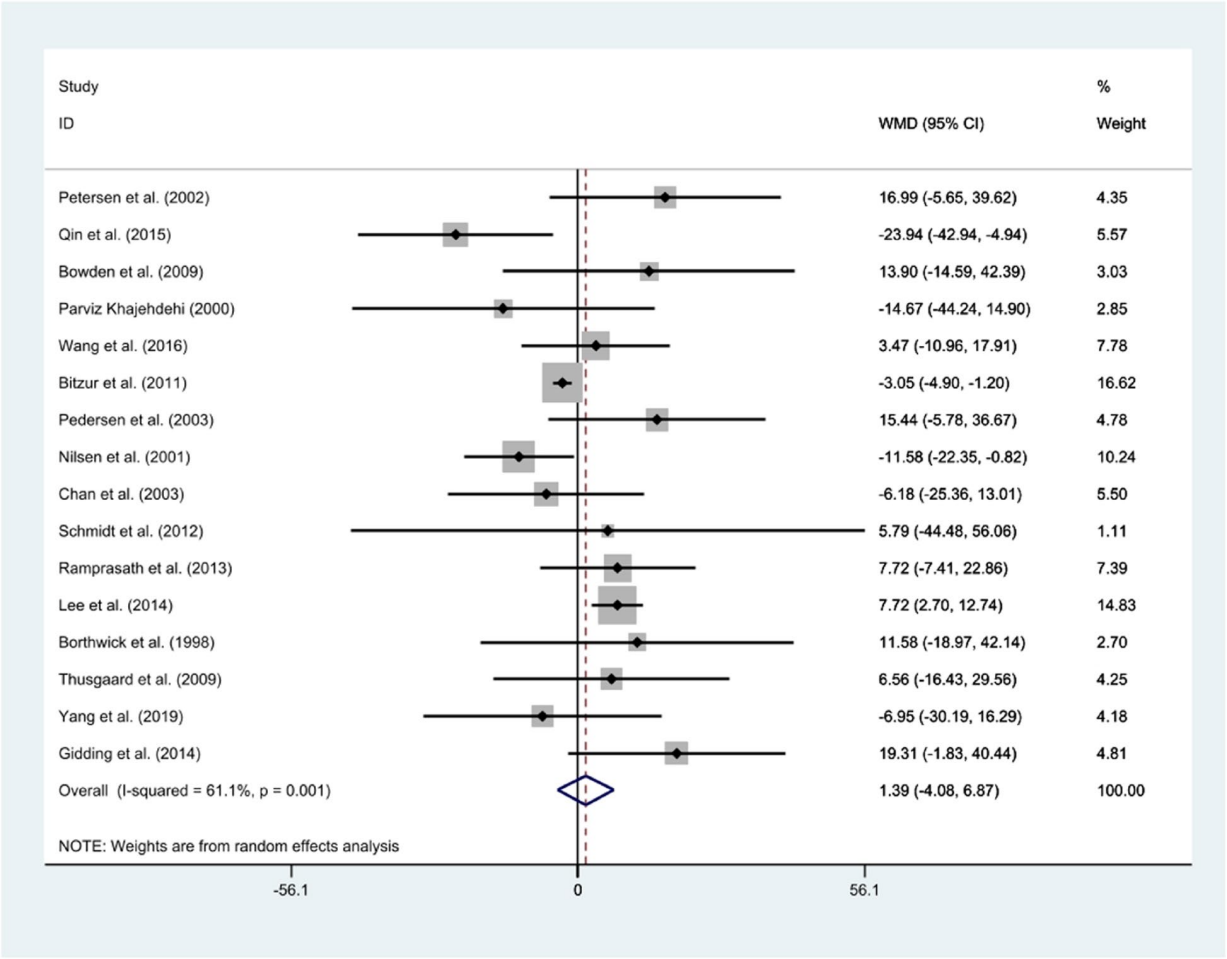
Forest plot of clinical trials investigating the comparison of fish oil supplements and corn oil effects on the serum TC

The combined results of the random-effects model showed no significant reduction in TC level following fish oil intake compared to corn oil (*WMD*: 1.39 mg/dl, 95% *CI*: − 4.08, 6.87, *P* = 0.001), which was identified by significant heterogeneity (*I*^2^ = 61.1%; *p* = 0.001) (Fig. 2). Subgroup analysis indicated no potential source of heterogeneity based on age, duration, health condition, or dose (Table 2). The sensitivity analysis for TC revealed that no individual study had a great impact on the overall effect size. Visual inspection of the funnel plot showed no evidence of publication bias (Fig. 3). Moreover, Egger’s test confirmed the same result (*P* = 0.253).

**Table 2:** Subgroup analysis of included randomized controlled trials in the systematic review and meta-analysis of comparison of canola oil and olive oil consumption on the serum lipid profile in adults

Variables	Subgroups	No. of trials	P-heterogeneity	*I*^2^ (%)	WMD (95% *CI*)	P_between_
TG		15				
	Age					0.00
	< 50	7	0.00	81.8	− 36.28 (− 52.20, − 19.46)	
	> 50	8	0.00	63.0	− 16.81 (− 34.51, 0.88)	
	Duration (week)					0.00
	≤ 60	7	0.00	94.2	− 26.54 (− 58.40, 6.19)	
	> 60	8	0.00	80.1	− 25.66 (− 47.78, − 2.65)	
	Dose					0.00
	< 4	5	0.70	0	− 14.15 (− 33.62, 5.30)	
	≥ 4	9	0.00	89.8	− 34.51 (− 53.09, − 15.92)	
	Health status					0.00
	Type 2 diabetes	4	0.00	78.3	− 34.68 (− 73.43, 4.07)	
	Dyslipidemia	4	0.00	88.4	− 40.14 (− 58.77, − 21.52)	
	Others	7	0.14	37.5	− 12.94 (− 30.52, 4.63)	
TC		16				
	Age					0.00
	< 50	7	0.05	51.0	− 0.85 (− 10.12, 8.42)	
	> 50	9	0.05	47.1	3.17 (− 4.17, 10.52)	
	Duration (week)					0.00
	≤ 60	7	0.36	8.1	7.66 (2.3, 12.99)	
	> 60	9	0.20	26.9	− 3.83 (− 9.50, 1.83)	
	Dose					0.05
	< 4	5	0.69	0	5.40 (− 5.12, 15.93)	
	≥ 4	10	0.02	53	− 0.94 (− 7.77, 5.89)	
	Health status					0.00
	Type 2 diabetes	4	0.69	0	8.03 (3.49, 12.56)	
	Dyslipidemia	5	0.25	25.3	0.52 (− 7.83, 8.88)	
	Others	7	0.08	45.3	− 5.01 (− 15.0, 4.98)	
HDL-c						
	Age					0.00
	< 50	6	0.18	33.4	1.77 (0.21, 3.33)	
	> 50	9	0	88.1	3.01 (− 0.82, 6.84)	
	Duration (week)					0.00
	≤ 60	6	0	81.5	1.29 (− 2.10, 4.67)	
	> 60	9	0	82.6	3.58 (0.48, 6.68)	
	Dose					0.00
	< 4	4	0.00	75.8	6.88 (− 0.80, 3.44)	
	≥ 4	10	0.02	52.5	1.91 (0.38, 3.44)	
	Health status					0.00
	Type 2 diabetes	4	0	87	3.20 (− 2.68, 9.07)	
	Dyslipidemia	5	0.11	45.8	1.64 (− 0.05, 3.33)	
	Others	6	0	84.9	3.47 (− 1.99, 8.94)	
LDL-c						
	Age					0.00
	< 50	7	0.01	62.1	3.42 (− 2.79, 9.63)	
	> 50	6	0.12	42.1	4.63 (− 2.20, 11.45)	
	Duration (week)					0.00
	≤ 60	6	0.29	17.8	9.21 (4.71, 13.70)	
	> 60	7	0.94	0	− 0.06 (− 1.67, 1.55)	
	Dose					0.02
	< 4	5	0.93	0	4.30 (− 3.67, 12.27)	
	≥ 4	7	0	70.1	2.48 (− 3.78, 8.73)	
	Health status					0.00
	Type 2 diabetes	3	0.25	27.1	9.69 (1.91, 17.47)	
	Dyslipidemia	4	0	78.4	2.65 (− 5.69, 11)	
	Others	6	0.70	0	0.67 (− 5.60, 6.93)

#### TG

**Fig. 4:**
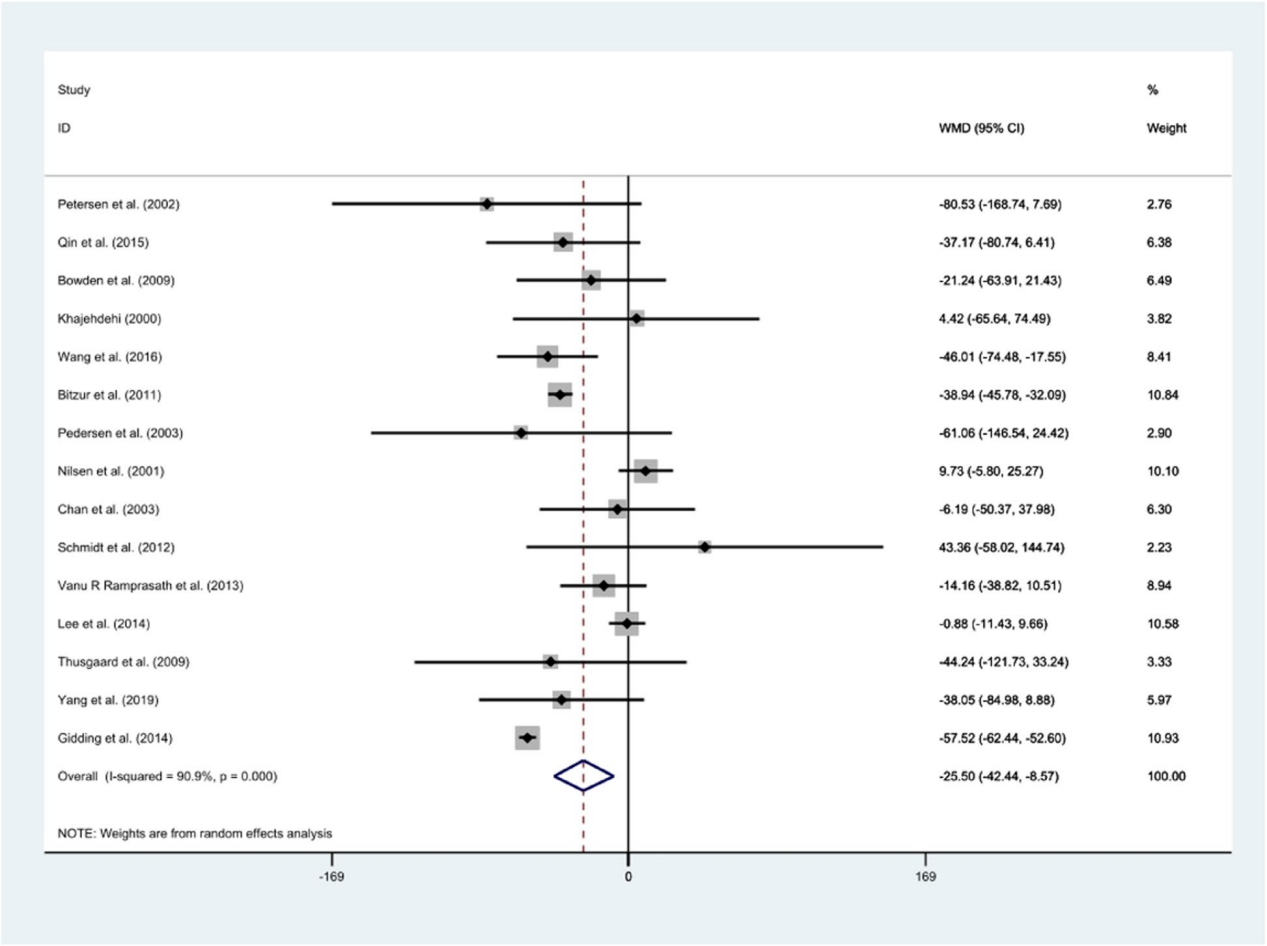
Forest plot of clinical trials investigating the comparison of fish oil supplements and corn oil effects on the serum TG

Pooling data from 15 studies, a significant reduction in TG levels (*WMD*: − 25.50 mg/dl, 95% *CI*: − 42.44, − 8.57, *P* = 0.000) was found after fish oil consumption than the controls. There was high heterogeneity between studies (*I*^2^ = 90.9%; *P* = 0.000) (Fig. 4). Age, dose, duration of intervention, and health status of subjects were considered as possible sources of heterogeneity. The reduction of TG was significant in studies done in participants aged ≤ 50 years (*WMD*: − 36.15 mg/dl, 95% *CI*: − 52.49, − 19.81), with dosage ≥ 4 g/day (*WMD*: − 34.36 mg/dl, 95% *CI*: − 52.83, − 15.89), duration > 9 weeks (*WMD*: − 25.39 mg/dl, 95% *CI*: − 47.76, − 3.01), and conducted among those with dyslipidemia (*WMD*: − 40.14 mg/dl, 95% *CI*: − 58.87, − 21.52). Overall meta-analysis result for LDL-c was not sensitive to individual studies. Assessment of publication bias by visual inspection of a funnel plot illustrated no sign of publication bias (Fig. 2). Egger’s test demonstrated no publication bias (*P* = 0.16).

#### HDL-c

**Fig. 5:**
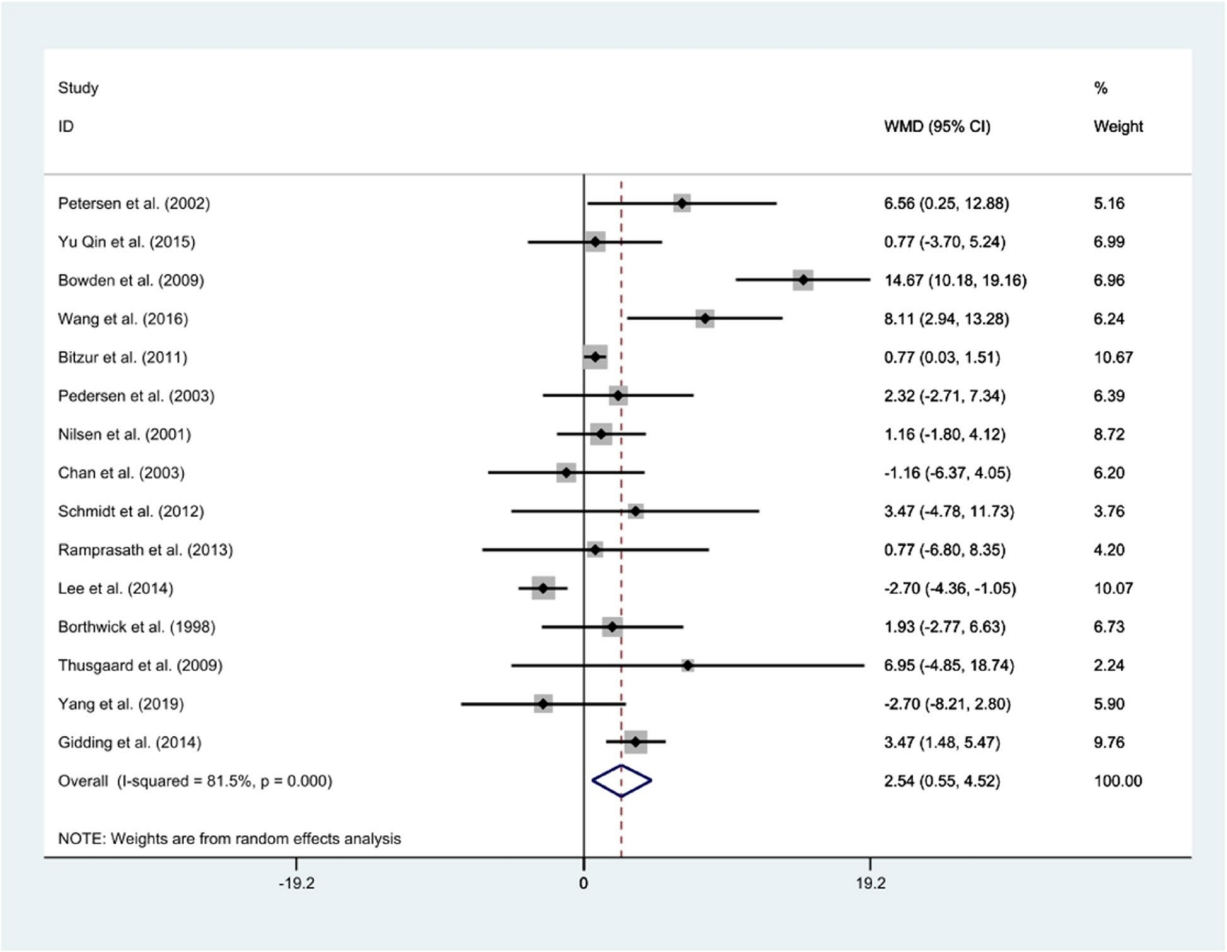
Forest plot of clinical trials investigating the comparison of fish oil supplements and corn oil effects on the serum HDL-c

The pooled estimate from the random-effect model performed on 15 studies showed that fish oil supplementation had a significant positive effect on the serum level of HDL-c in comparison to corn oil (*WMD*: 2.54 mg/dl, 95% *CI*: 0.55, 4.52), with significant heterogeneity (*I*^2^ = 81.5%; *P* = 0.000) (Fig. 5). However, subgroup analysis showed that age, dose, health status, and duration were potential sources of heterogeneity (Table 2). The sensitivity analysis revealed that no study had a significant impact on the overall effect size of HDL-c. Evaluation of publication bias by visual inspection of a funnel plot displayed no evidence of publication bias among the included studies (Fig. 2). The same finding was also concluded by Egger’s regression test (*P* = 0.19).

#### LDL-c

**Fig. 6:**
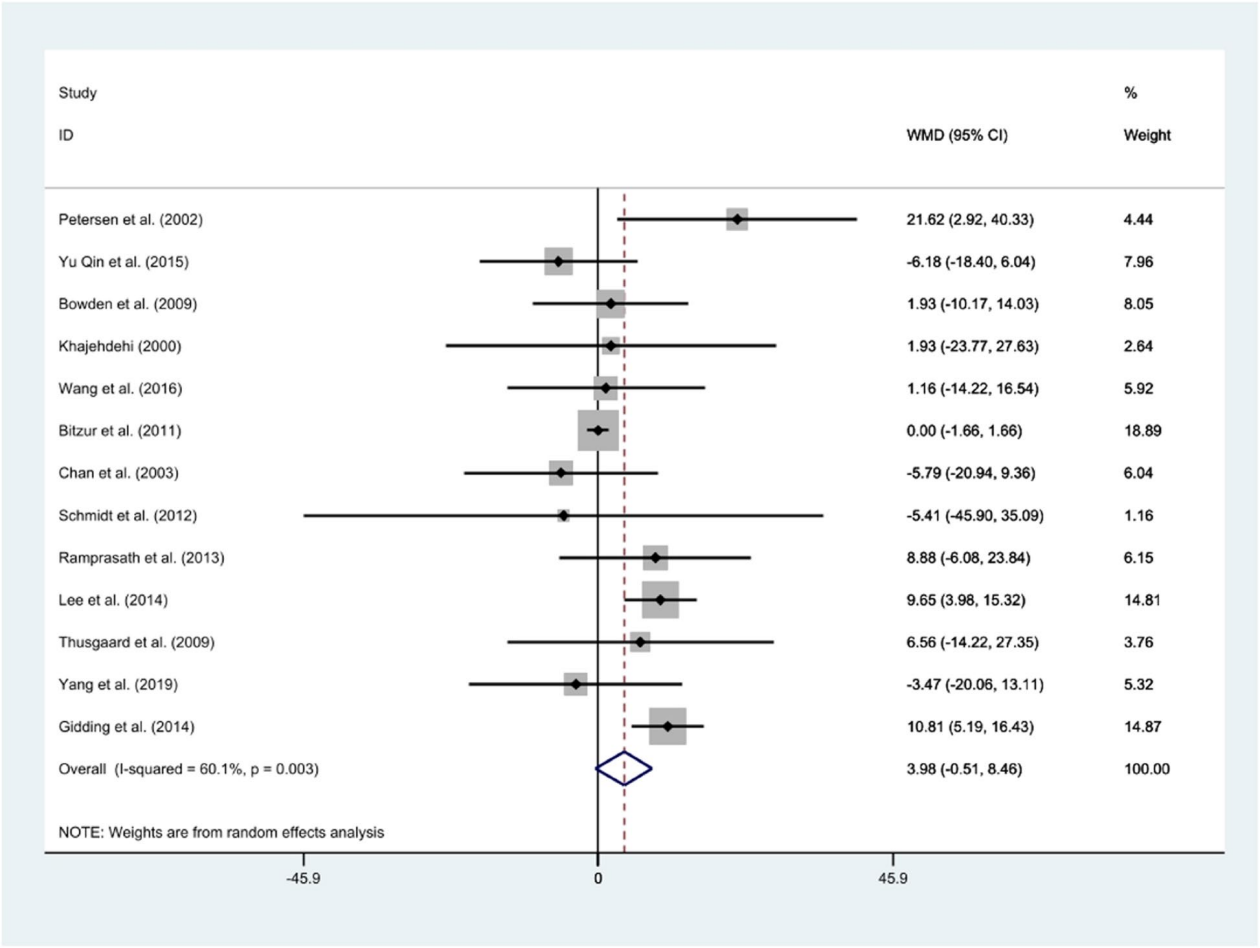
Forest plot of clinical trials investigating the comparison of fish oil supplements and corn oil effects on the serum LDL-c

The effect of fish oil supplements on LDL-c concentrations was examined in 13 clinical trials. The analysis revealed no significant reduction in the LDL-c concentration following fish oil consumption compared to control groups (*WMD*: 3.98 mg/dl, 95% *CI*: − 0.51, 8.46) (Fig. 6). There was high heterogeneity between the effect sizes of the included studies (*I*^2^ = 60.1%; *P* = 0.003). The sensitivity analysis provided no evidence of the impact of an individual study on the overall result. Subgroup analysis indicated no potential source of heterogeneity based on age, dosage, health condition, or study duration (Table 2). Visual inspection of the funnel plot (Fig. 2) and further assessment using the Egger test did not suggest evidence of publication bias (Egger test: *P* = 0.301).

## Discussion

This systematic review and meta-analysis evaluated 16 clinical controlled trials that examined the effects of fish oil supplements on lipid profiles compared to corn oil. Our meta-analysis results proved that fish oil significantly decreased TG and increased HDL-c levels compared to corn oil. However, no significant associations were found in other lipids, including TC and LDL-c. Our findings are similar to the results of a previous meta-analysis on the effect of fish oil supplementation on lipid levels among patients with type 2 diabetes. Gao et al. (2020) found that fish oil supplementation reduced TG level by − 35.39 (95% *CI*: − 46.89, − 24.77, *I*^2^ = 0%, *p* < 0.05) and increased HDL-c level by 8.10 (95% *CI*: 1.93, 14.28, *I*^2^ = 37.1%, *p* < 0.05), whereas TC and LDL-c were not significantly affected [41]. Almost in line with our study, the meta-analysis carried out by Wu et al. (2021) which included 12 RCTs reported a significant decrease in serum triglyceride (*WMD*: − 21.23 mg/dl, 95% *CI*: − 35.39 to − 7.07, *P* = 0.004) but had no significant effects on TC, LDL-c, or HDL-c in subjects with overweight after taking fish oil compared to the control group [42]. Similar to our result, another meta-analysis based on 13 RCTs evaluating the effects of fish oil supplementation on serum lipid profile in dialysis patients showed a significant improvement in TG, TC, and HDL-c levels compared with the control group, while no changes were seen in LDL-c levels. In contrast, they reported a decrease in TC concentration [43]. The different control groups that were included in the study of Zhu et al. (2014) without subgroup analysis but were excluded in the present study can be considered a reason for the significance of the TC results compared to our work. Another reason may be the absence of subgroup analyses based on specific oils that were included in the meta-analysis as a control group in order to know more about the effects of fish oil on the lipid profile. Subgroup analysis was conducted based on age, dose, and duration of the articles related to fish oil and corn oil, unlike the meta-analysis by Zhu et al. (2014) and Wu et al. (2021), in which subgroup analysis was performed on articles related to fish oil supplementation with patients receiving different control oils. Moreover, in the current study, unlike the mentioned meta-analyses, the age and different health statuses of participants have also been calculated.

Fish oil is derived from the tissues of cold-water oily fish, such as mackerel, tuna, herring, sardines, and salmon [44]. It has become one of the most widely consumed supplements. Fish oil is a rich source of omega-3 PUFAs, mainly EPA and DHA [45]. On the other hand, corn oil is a kind of refined vegetable oil that is valued for its cooking properties, and its content, especially linoleic acid and phytosterols, is responsible for its health benefits. In addition, it has been demonstrated that, despite having a high PUFA content, there is a lower amount of SFAs in corn oil than in other vegetable oils, including soybean and cottonseed oil [46]. Ghobadi et al. (2019) reported that a diet rich in PUFA and low in SFA can improve lipid profiles [47]. Many mechanisms of action of PUFAs have been proposed for their effects on blood lipids. Fatty acid composition, such as EPA and DHA, is one of the underlying mechanisms for the effect of fish oil supplements on blood lipids [48]. Furthermore, available research evidence from studies shows that omega-3 PUFA alters the function of cellular phospholipids and lowers triglycerides by inhibiting the phosphatidic acid phosphatase [49]. Previous studies have shown that the prescription of fish oil supplements containing omega-3 fatty acids can reduce serum TG levels. For instance, Tummala et al. (2019) reported that omega-3 fatty acid esters at high doses reduced fatty acid synthesis from carbohydrates and lowered triglycerides, therefore leading to a decrease in cardiovascular diseases [50]. Another study conducted by Shearer et al. (2012) demonstrated that n-3 FA has contributed to a reduction in triacylglycerol synthesis due to decreased diacyl-glycerol acyltransferase activity, the necessary enzyme associated with the production of triglyceride-rich lipoproteins in the liver [51]. Additionally, n-3 FA treatment may inhibit the assembly of apolipoprotein-B100 and VLDL-c particles and consequently cause a reduction in TG levels [52, 53]. In our study, the serum content of TG was reduced following the intervention of fish oil supplements compared to corn oil.

This meta-analysis has some advantages. The current study is the first meta-analysis on the comparison of fish oil supplements and corn oil consumption on the serum lipid profile. The comprehensive evidence search was so precious and performed without language or time restrictions to find all relevant publications. On the one hand, only randomized controlled trials were included in the analysis. Also, the statistical test for the determination of publication bias was non-significant. However, this study has some limitations. Participants in the included studies had different health statuses, including T2DM, diabetes, hypertension, dyslipidemia, HIV, kidney failure, overweight, and healthy subjects. Therefore, the extracted data showed different baseline levels of lipid profiles; however, we conducted a subgroup analysis. Moreover, finding the sources of heterogeneity is another concern that was not completely resolved by subgroup and sensitivity analyses.

## Conclusion

In summary, compared with the control group, combined findings from 16 eligible clinical trials showed that dietary intake of fish oil supplements containing n-3 FA significantly decreased and increased TG and HDL-c, respectively. However, no significant effect was observed on TC and LDL-c variables, which are CVD risk factors. Thus, fish oil supplements might be efficient in reducing the mentioned serum lipids and preventing heart disease. Additional studies with larger sample sizes and different doses are needed to confirm our findings.
